# COHORT DIFFERENCES IN COGNITIVE AGING: THE ROLE OF PERCEIVED WORK ENVIRONMENT

**DOI:** 10.1093/geroni/igz038.091

**Published:** 2019-11-08

**Authors:** Gizem Hueluer, Nilam Ram, Sherry L Willis, K Warner Schaie, Denis Gerstorf

**Affiliations:** 1 University of Zurich, Zurich, Switzerland, Switzerland; 2 Pennsylvania State University, University Park, Pennsylvania, United States; 3 University of washington, Seattle, Washington, United States; 4 University of Washington, Seattle, Washington, United States; 5 Humboldt University, Berlin, Berlin, Germany

## Abstract

Studies of historical change on cognitive aging generally document that later-born cohorts outperform earlier-born cohorts on tests of fluid cognitive performance. It is often noted how advances in educational attainment contribute to this finding. Over the last century, work demands and characteristics have changed profoundly, with shifts from a manufacturing to service and technical economy. We used data from the Seattle Longitudinal Study to compare trajectories of cognitive change between earlier-born (1901-1938) and later-born cohorts (1939-1966). Our findings show that (a) later-born cohorts had higher levels of performance on most cognitive tasks and exhibited less decline in word fluency, (b) had more enriched perceived work environment as indicated by higher levels of worker control and innovation, with no cohort differences in work autonomy (c) these experiences were associated with higher levels of cognitive performance independent of education and consistently across cohorts. We discuss potential mechanisms underlying these associations.

